# Structural Insights into Catalytic Versatility of the Flavin-dependent Hydroxylase (HpaB) from *Escherichia coli*

**DOI:** 10.1038/s41598-019-43577-w

**Published:** 2019-05-08

**Authors:** Xiaolin Shen, Dayong Zhou, Yuheng Lin, Jia Wang, Shuaihua Gao, Palani Kandavelu, Hua Zhang, Ruihua Zhang, Bi-Cheng Wang, John Rose, Qipeng Yuan, Yajun Yan

**Affiliations:** 1 Beijing Advanced Innovation Center for Soft Matter Science and Engineering, Beijing University of Chemical Technology, Beijing, 100029 China; 2 State Key Laboratory of Chemical Resource Engineering, Beijing University of Chemical Technology, Beijing, 100029 China; 3 Department of Biochemistry & Molecular Biology, The University of Georgia, Athens, Georgia 30602 USA; 4 School of Chemical, Materials and Biomedical Engineering, College of Engineering, The University of Georgia, Athens, GA 30602 USA

**Keywords:** Structure determination, Metabolic engineering

## Abstract

4-Hydroxyphenylacetate 3-hydroxylase (EcHpaB) from *Escherichia coli* is capable of efficient *ortho*-hydroxylation of a wide range of phenolic compounds and demonstrates great potential for broad chemoenzymatic applications. To understand the structural and mechanistic basis of its catalytic versatility, we elucidated the crystal structure of EcHpaB by X-ray crystallography, which revealed a unique loop structure covering the active site. We further performed mutagenesis studies of this loop to probe its role in substrate specificity and catalytic activity. Our results not only showed the loop has great plasticity and strong tolerance towards extensive mutagenesis, but also suggested a flexible loop that enables the entrance and stable binding of substrates into the active site is the key factor to the enzyme catalytic versatility. These findings lay the groundwork for editing the loop sequence and structure for generation of EcHpaB mutants with improved performance for broader laboratory and industrial use.

## Introduction

Hydroxylation of carbon atom is an important chemical reaction involved in many chemical and biological processes, such as environmental remediation and synthesis or biosynthesis of various chemicals and biological molecules^1–3^. Particularly, a variety of high-value natural products, such as terpenes, alkaloids, unnatural amino acids and phenylpropanoids involve hydroxylation reactions in their synthesis^4–8^. However, stereospecific hydroxylation at carbon on a target molecule can be a quite challenging task for synthetic chemistry, since excitation of the specific carbon atom usually leads to low regio-/stereo-selectivity and low catalytic efficiency; and often requires the protection and de-protection of other chemical groups, which could be laborious and inefficient. Alternatively, synthesis of hydroxylated compounds by biological hydroxylation becomes an attractive approach owing to numerous available hydroxylases capable of hydroxylating specific carbon atoms in organisms. For instance, cytochrome P450 superfamily hydroxylases are such enzymes that have been widely used for regio-specific hydroxylation of carbon atoms to synthesize various value-added products^9,10^. With the understanding of their key structural characteristics and their implications in substrate specificity and catalytic activity, these enzymes have been extensively engineered to accept non-natural substrates for chemoenzymatic applications with a series of protein engineering toolkits and methods developed^11–13^. However, their low expression level in microorganisms, requirement of reductase partners and intrinsic low enzyme activity limit their broad use in large-scale settings^14^. To overcome these hurdles, hydroxylases with better catalytic properties and flexibility are highly desired. Similar to CYP450 hydroxylases, flavin adenine dinucleotide (FAD) dependent hydroxylases are also a protein superfamily, which are capable of hydroxylation reactions in organisms. These enzymes usually contain a NAD(P)H binding domain and an FAD binding domain; and utilize NAD(P)H as cofactor and FAD as prosthetic group to hydroxylate their substrates, which provide faster substrate turnover than CYP450 enzymes^14,15^. However, it is difficult to engineer their enzymatic activity and substrate spectrum due to the less understanding of their structure-activity relationship^16,17^.

As an FAD-dependent hydroxylase, 4-hydroxyphenylacetate 3-hydroxylase (HpaB) is able to *ortho*-hydroxylate phenylpropanoids in a molecular chaperon-independent manner and has broad substrate specificity and high catalytic activity^14,18^. HpaB has been identified from *Thermus thermophilus*^19^, *Acinetobacter baumannii*^20^, *Pseudomonas putida*^21^, *Geobacillus sp*. PA-9^22^, *Klebsiella pneumoniae*^23^ and *Escherichia coli*^24^. Among them, the HpaBC complex from *E. coli* (EcHpaBC) has outstanding activities and a broad substrate spectrum. EcHpaBC has two components, EcHpaB and EcHpaC. EcHpaB is an FAD-dependent monooxygenase catalyzing the hydroxylation reaction; while EcHpaC as a NAD(P)H-flavin utilizing oxidoreductase is characterized as an electron donor transferring an electron to FAD^+^ regenerating FADH_2_ in EcHpaB^17,24^. EcHpaB natively catalyzes the *ortho*-hydroxylation of 4-hydroxyphenylacetate (4HPA) into 3,4-dihydroxyphenylacetate (3,4-DHPA), which initiates 4HPA degradation^25^. More interestingly, we found that EcHpaB is also capable of hydroxylating a series of phenylpropanoids to generate a wide range of natural products^14^. For example, in our previous study, 3.5 g/L of *p*-coumaric acid, which is structurally similar to 4HPA, was hydroxylated into caffeic acid (3.82 g/L) with a conversion ratio close to 100% in whole-cell catalysis by this hydroxylase^26^. Umbelliferone is another substrate that can be hydroxylated by EcHpaB. When 2 g/L of umbelliferone was fed, 2.7 g/L of esculetin was generated with a yield of up to 98%^14^. Resveratrol as a larger substrate than *p*-coumaric acid and umbelliferone in molecular size, has also been hydroxylated into piceatannol at a titer of 1.2 g/L^14^. EcHpaB even has the ability to catalyze another two larger substrates, naringenin and afzelechin, into their corresponding *ortho*-hydroxylated products, eriodictyol and catechin, although the efficiency was much less. The highest titers were reported at 62.7 mg/L and 34.7 mg/L in whole-cell catalysis studies, respectively^18^. These results indicate that EcHpaB has a broad substrate spectrum for the hydroxylation of a variety of phenolic compounds. Interestingly, the HpaB from *T. thermophilus* HB8* (TtHpaB) demonstrates much stricter substrate specificity with low catalytic efficiency^26^. The differences in substrate spectrum and catalytic properties of these two enzymes provide a unique opportunity to identify the key structural characteristics that render the catalytic flexibility to these enzymes. This knowledge would constitute a foundation to unlock the potential of these enzymes as versatile biocatalyst for use in biological and/or chemical processes through protein engineering efforts, which is of great practical value.

In this study, we solved the crystal structure of EcHpaB apoenzyme for the first time by X-ray crystallography (Table S2). Structural alignment of EcHpaB and TtHpaB revealed a distinct sequence and structural difference in the loop connecting strands β32 and β33 and covering the entrance of the catalytic site, which consists of eleven amino acids. To further understand the role of the loop in the catalytic properties and flexibility of EcHpaB, we performed structure-based mutagenesis to introduce perturbations to the sequence and structure of this loop, which generated five mutants. We further used a set of natural and non-natural substrates with gradually increased molecular sizes to survey the catalytic properties changes among these mutants through *in vivo* and *in vitro* enzymes assays. These efforts led to the identification of a mutant (XS6) demonstrating increased activity towards non-natural larger substrates resveratrol and naringenin. To understand the mechanistic basis of these changes, we next elucidated the crystal structure of this mutant. The further sequence and structural analysis suggested that the enlarged entrance of substrate-binding pocket and improved flexibility of the loop structure caused by the changes in the amino acid sequence and the corresponding changes in the loop structure may play important roles in the improved catalytic versatility of the mutant. Overall, these studies provide structural insights into the substrate preference of this FAD-dependent hydroxylase and suggest a key sequence and structural feature for protein engineering efforts aiming at altering the catalytic properties and versatilities of these type of enzymes for broader laboratory and industrial use.

## Results

### The overall EcHpaB structure

**Figure 1:**
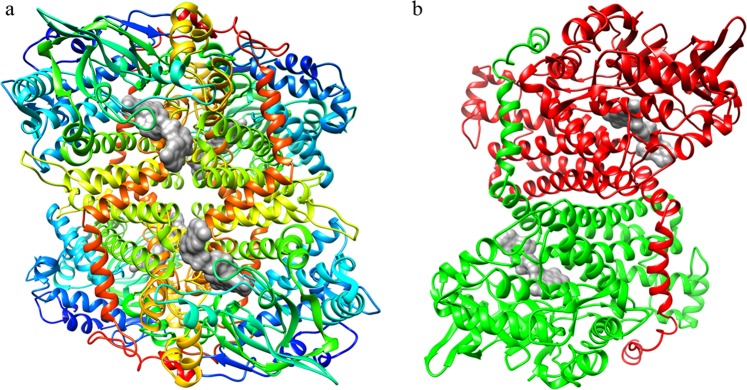
(**a**) A ribbon drawing of the EcHpaB tetramer found in the crystallographic asymmetric unit. The four HpaB monomers colored blue to red based on sequence position (N-terminal residues depicted in blue and C-terminal residues depicted in red). (**b**) A ribbon drawing of the EcHpaB dimer, chains A (red) and B (green), showing the C-terminal helical arms interacting with it’s dimer partner. The putative FAD cofactor (gray), based on the overlapped TtHpaB active site model is included to indicate the position of the active site pocket.

EcHpaB is an (α_2_)_2_ tetramer (Fig. 1a), the model consists of residues 2–519 for each of the four-polypeptide chains in the crystallographic asymmetric unit. The N-terminal (MHHHHHHHK) purification tags (residues −7 to 0), Met 1 and the Lys 520 for each chain were not observed in the electron density maps and were excluded from the final model. The tetramer can best be described as dimer of homodimers with chains A and B forming one dimer and chains C and D forming the other (Fig. 1b). A major feature of the EcHpaB dimer are the C-terminal tails formed by residues Leu 456 to Leu 519 (helices α27, α28, α29 and α30. Fig. S1) which extends from one EcHpaB monomer and wraps around its counterpart. Nine intermolecular hydrogen bonds between the arm and its counterpart also help stabilize the dimer. The overall EcHpaB structure contains 30 helices and 16 β strands, which make up five β sheets (Fig. S1). Sheet β1 is a six-stranded mixed β sheet composed of strands β12 (denoted as sheet number and strand ID, PDB ladder ordering), β11, β16, β15 and β13. Sheet β2 is a two-stranded antiparallel β sheet composed of strands β21 and β22. Sheet β3 is a four-stranded mixed β sheet composed of strands β31, β32, β33 and β34. Sheet β4 is a two-stranded antiparallel β sheet composed of strands β41 and β42. Sheet β5 is a two-stranded antiparallel β sheet composed of strands β51 and β52.

**Figure 2:**
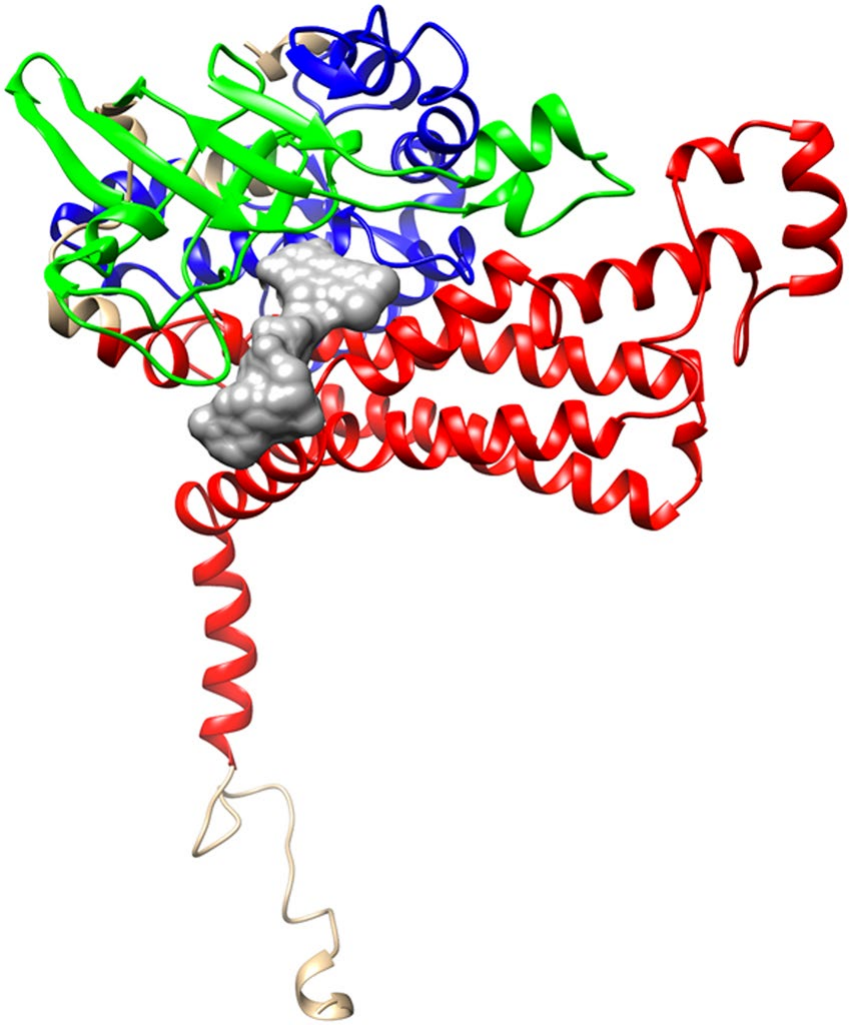
A ribbon drawing of EcHpaB monomer (chain A) colored according to the CATH structural domains that make up the structure^27^. Here domains are colored as follows: (blue) CATH domain 1u8vA01 residues 12–148; (green) CATH domain 1u8vA02 residues 155–281; (red) CATH domain 1r2jA03 residues 296–489. The modeled FAD cofactor (gray) is included to indicate the position of the active site pocket.

EcHpaB exhibits three-domain architecture found in other FADH_2_-dependent HpaB structures (Fig. 2)^16^: an N-terminal mainly helical domain residues Phe 14 to Glu 148, which contains helices α2 to α10 and β strands β11, β12, β21 and β22; a mainly beta middle domain residues His 155 to Ile 281, which contains helices α11 to α17 and β strands β13 to β16 and β31 to β34; and a mainly helical C-terminal domain residues Gly 296 to Val 489, which contains helices α18 to α29 and β strands β41, β42, β51 and β52^27^. The putative FAD and substrate binding sites lie within a grove between the three domains.

### The EcHpaB active site

In order to confirm the spatial geometry of the EcHpaB active site a theoretical model of EcHpaB with bound FAD and the 4HPA substrate was generated using the crystal structure of the active TtHpaB enzyme containing both cofactor and substrate (PDB entry 2YYJ) as template (Note: In the following discussion TtHpaB residues are identified in italics while EcHpaB residues are identified in normal typeface).

In the TtHpaB structure, the 4HPA substrate is anchored in the binding site by hydrogen bonding of its phenol hydroxyl group to residues *Arg 100*, *Tyr 104* and *His 142*, which are structurally conserved in EcHpaB (Arg 113, Tyr 117 and His 155). The carboxyl tail of the substrate is anchored in the binding site *via* hydrogen bonding to the side chain (OH) of *Ser 197* and to the main chain (NH) and side chain (OH) of *Thr 198*. Both residues are located on the β32-β33 loop. Since this loop is out of place in the EcHpaB apo-structure, modeling of the EcHpaB active site included replacing the β32-β33 loop (residues 207 to 217) in the EcHpaB structure with the β32-β33 loop (residues 193 to 213) from the TtHpaB 2YYJ structure and adding FAD and the 4HPA substrate from the TtHpaB 2YYJ structure. The resulting model showed that most of the substrate binding interaction are maintained in the EcHpaB structure with the exception of *Thr 198*, which is replaced by Ala 211 resulting in a loss of a side chain hydrogen bond.

Several other potential HpaB substrates of increasing complexity were also modeled into the enzyme active site. These include *p*-coumaric acid, tyrosine, umbelliferone, resveratrol and naringenin. In all cases the crude modeling suggested that the EcHpaB active site could accommodate these substrates in part owing to the plasticity of the β32-β33 loop and the observation that main chain hydrogen bonding to loop residues played a key role in positioning the substrate in the active site. These studies also suggested that mutation of the β32-β33 loop residues could affect catalytic performance. However, it must be stressed that these modeling results only show potential binding ability, which must be confirmed via either binding studies or the determination of the active FAD bound structure with bound substrate.

### Structural and catalytic characteristic comparison of EcHpaB and TtHpaB

**Figure 3:**
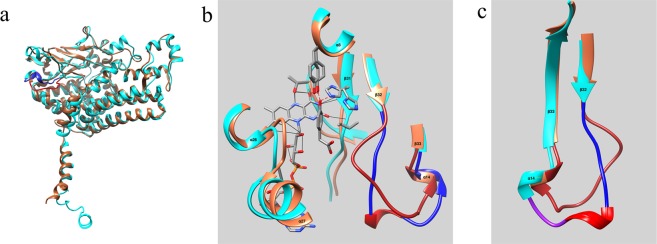
(**a**) A superposition of the apo structures of the EcHpaB (cyan) and TtHpaB (coral). The β32-β33 loop is highlighted in dark blue (EcHpaB) and red (TtHpaB). (**b**) A view of the EcHpaB (cyan) active site. Enzyme-putative FAD hydrogen bonds are denoted by thin black lines while enzyme substrate hydrogen bonds are denoted by red dashed lines. The β32-β33 loop is highlighted in dark blue for EcHpaB and dark coral for TtHpaB. (**c**) A comparison of the EcHpaB β32-β33 loop structure with that of TtHpaB structures. Blue indicate section I. Red indicate section II and purple indicate section III.

As described above, TtHpaB, which has three domains like EcHpaB, also catalyzes the conversion of 4HPA to 3,4-DHPA^16^. The amino acid sequences of EcHpaB and TtHpaB were compared using the Basic Local Alignment Search Tool (BLAST) at the National Center for Biotechnology Information (NCBI) website. EcHpaB is composed of 520 amino acids while TtHpaB contains 481 amino acids. BLAST analysis reveals that the amino acids sequence identity between these two enzymes is only 30%. Interestingly, the low-homology sequences of EcHpaB and TtHpaB enzymes results in highly similar three-domain architecture and 3-dimensional structure (Fig. 3a). Considering the contrast between sequence and structural homology exhibited by the two enzymes, the catalytic properties of the two enzymes were compared.

**Figure 4:**
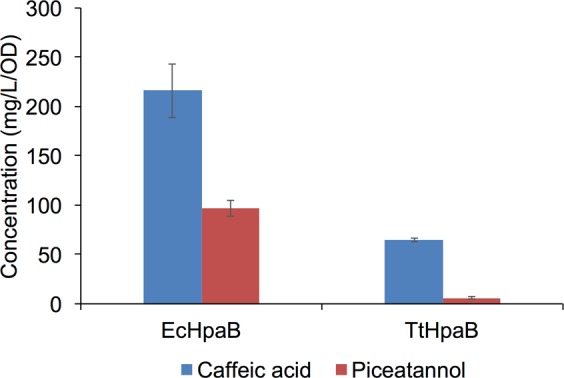
Results of whole cell bioconversion experiments using EcHpaB and TtHpaB with *p*-coumaric acid and resveratrol as substrates. All data points are reported as mean ± s.d. from three independent experiments.

In the analysis, *p*-coumaric acid and resveratrol were used as the substrates to test the *in vivo* catalytic activity and substrate specificity of TtHpaB and EcHpaB. Interestingly, EcHpaB demonstrated higher activity towards *p*-coumaric acid and resulted in final titer of 216.18 mg/L/OD caffeic acid after 12 h, while TtHpaB gave a lower titer (64.75 mg/L/OD caffeic acid) (Fig. 4). The same trend was observed for the conversion of resveratrol. The titer of piceatannol afforded by EcHpaB reached 96.65 mg/L/OD, while only 5.77 mg/L/OD piceatannol was produced by TtHpaB (Fig. 4). When using the conversion rate of *p*-coumaric acid as a reference (100%) to quantify the catalytic efficiencies of EcHpaB and TtHpaB towards resveratrol, the efficiencies decreased by 2.24 and 11.23 folds, respectively. These results indicate that the tolerance of EcHpaB towards resveratrol, a larger substrate is higher than that of TtHpaB. In order to find out the differences in EcHpaB and TtHpaB that may lead to their different catalytic characteristics, we performed the structure alignment of EcHpaB and TtHpaB. As shown in Fig. 3a, The structures of the EcHpaB and TtHpaB apoenzymes can be superimposed^28^ giving an RMSD of 1.2 Å for 405 Cα pairs, which indicates that the overall structure of the two enzymes is highly similar with the major differences occurring in the β32-β33 loop (residues 207–217). This loop has been shown to undergo a significant conformational change upon FAD and ligand binding in TtHpaB^16^. As described above, modeling studies have shown that active sites of the EcHpaB and TtHpaB enzymes are also similar (Fig. 3b). However, based on the secondary structure prediction, the EcHpaB β32-β33 loop appears to be segmented into three sections, two random coils were separated by a Type IV β-turn (Fig. 3c). In contrast, the corresponding loop in TtHpaB is a simple random coil. Thus, we deduced that the distinct differences between the β32-β33 loop structures in EcHpaB and TtHpaB enzymes may result in their different catalytic characteristics.

### Perturbation of the β32-β33 Loop to understand the enzyme catalytic properties

As mentioned above, the β32-β33 loop can be divided into three sections according to the secondary structure. Section I consists of amino acids 207–211 (GlyPheGlySerAla) forms a random coil. Section II contains amino acids 212–214 (GlnValMet), forms a Type IV β-turn. Section III consists of amino acids 215–217 (GlyGluAsn) that forms another random coil (Table 1 and Fig. 3c). To gain further insight into the impacts of the β32-β33 loop sequence and structure on enzyme catalytic characteristics, the amino acid sequence in this loop was altered intentionally to change the size and steric hindrance of these residues and/or the loop secondary structure.

**Table 1:** The amino acid sequences of the target loop used in this study.

Name of mutants	Amino acid sequences of the target loop
Wildtype HpaB	GlyPheGlySerAlaGlnValMetGlyGluAsn
XS2	**MetAlaMetValAsp**GlnValMetGlyGluAsn
XS3	GlyPheGlySerAlaGlnValMet**AlaMetVal**
XS4	**MetAlaMetValAsp**GlnValMet**AlaMetVal**
XS5	**MetAlaMetValAspGlySerGlyAlaMetVal**
XS6	**GlySerGlySerAspLeuGlySerGlySerAsp**

Bold fonts indicate the amino acid mutations.

**Figure 5:**
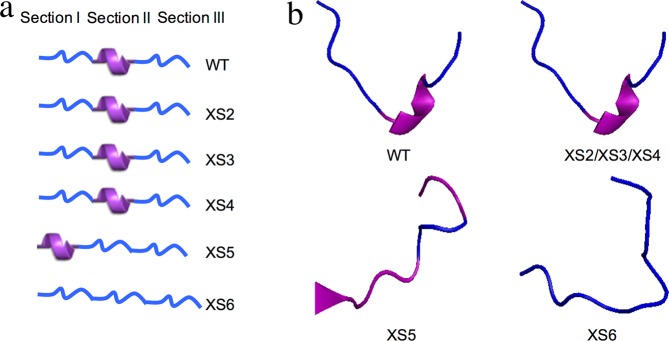
Diagrams of the loop structures. (**a**) The expected secondary structures. (**b**) model predicted secondary structures.

**Figure 6:**
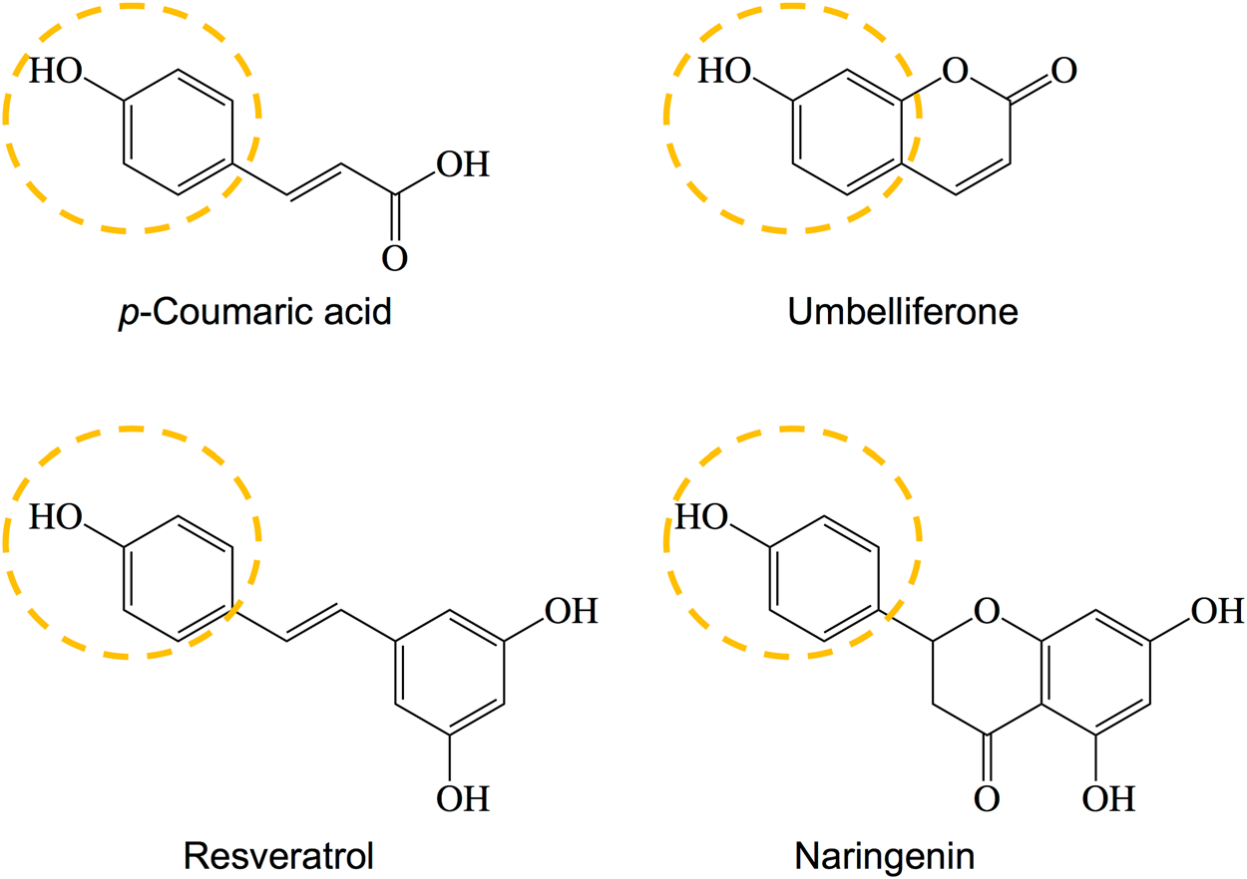
The chemical structures of *p*-coumaric acid, umbelliferone, resveratrol and naringenin. The yellow cycle indicated the phenol “head” which is recognized by EcHpaB.

First, we designed and created mutant XS2 by changing the amino acid residues of section I into larger ones while not changing the secondary structure of the loop (Table 1). Swiss-Model suggested that introduction of these mutations did not change the original secondary structure and the flexibility of the loop (Fig. 5a,b). In our previous study, the catalytic activities of wild type EcHpaB varied over a wide range depending on the substrate molecular size when employing *p*-coumaric acid, umbelliferone, resveratrol and naringenin as the substrates^14^. As shown in Fig. 6, these four molecules share the same phenol “head” motif which is recognized by EcHpaB but have different “tails” leading to increased molecular size. The catalytic capacity of EcHpaB decreased with the increased substrate molecular size. For the smallest substrate *p*-coumaric acid, the conversion ratio reached to 100%. While, for the largest substrate naringenin, only 11.1% was converted to eriodictyol^14^. EcHpaB have distinct catalytic capacities towards those substrates. Thus, we employed these four substrates to survey the catalytic characteristics of all the EcHpaB mutants in this study. As shown in Table 2, mutant XS2 showed decreased activity towards all four substrates. Especially, *K*_m_ value towards *p*-coumaric acid was increased to 387.9 μM, as compared to the wild type EcHpaB (137.6 μM), and its *k*_cat_ value was decreased by 2 folds. These results indicated that the introduced mutations in section I negatively affected the catalytic efficiency of EcHpaB, probably due to that the larger amino acid residues introduce or enhance the steric hindrance in this region and lead to a decreased apparent substrate binding affinity. Next, we designed and generated mutant XS3 by replacing the original amino acids in section III with the amino acids having larger residues (Table 1 and Fig. 5a). Swiss-Model indicated that the secondary structure of the loop in XS3 is same to that of the wild type enzyme (Fig. 5b). As shown in Table 2, compared with wild type EcHpaB, the specificity constant values (*k*_cat_/*K*_m_) of mutant XS3 decreased slightly towards *p*-coumaric acid and umbelliferone. The *K*_m_ values of mutant XS3 towards *p*-coumaric acid (235.6 μM) and umbelliferone (266.2 μM) increased by 1.7 folds and 1.2 folds, respectively. However, the *k*_cat_ value of XS3 towards (14.8 min^−1^) umbelliferone decreased by almost 2 folds. Interestingly, the specificity constant values (*k*_cat_/*K*_m_) toward resveratrol and naringenin did not change significantly. Those results demonstrated that introduction of the mutations to section III region of the loop appeared to have less impact on enzyme activity towards larger substrates. Following these, mutant XS4 was created by incorporating the mutations in both XS2 and XS3 (Table 1). The secondary structure of the loop in XS4 were still kept unchanged (Swiss Model, Fig. 5b). Surprisingly, the introduction of more mutations to the loop did not further decrease the catalytic activity (Table 2). Particularly, compared with XS2 and XS3, the specific constant values (*k*_cat_/*K*_m_) of XS4 towards *p*-coumaric acid and umbelliferone were even higher. When resveratrol and naringenin were used as substrates, these values fell in between those of XS2 and XS3. The catalytic capability of XS4 kept same with wild type EcHpaB, illustrating that even amino acid residues of both section I and section III were changed to larger ones, the loop has sufficient flexibility to buffer their effects on the catalytic characteristics. The differences in catalytic capability among XS2, XS3 and XS4 revealed that perturbation of the amino acids sequence in the loop have influence on catalytic properties of the enzyme even the loop secondary structure did not change. Additionally, the activities of all three mutants were largely remained even with extensive modifications introduced to the loop sequence, which indicates that this loop has great flexibility and plasticity to maintain enzyme activity and can be an excellent target for protein engineering efforts to alter catalytic characteristics of the enzyme.

**Table 2:** Kinetic parameters of EcHpaB and its variants toward different substrates.

	p-Coumaric acid			Umbelliferone			Resveratrol			Naringenin		
	*K*_m_ μM	*k*_cat_ min^−1^	*k*_cat_/*K*_m_ s^−1^mM^−1^	*K*_m_ μM	*k*_cat_ min^−1^	*k*_cat_/*K*_m_ s^−1^mM^−1^	*K*_m_ μM	*k*_cat_ min^−1^	*k*_cat_/*K*_m_ s^−1^mM^−1^	*K*_m_ μM	*k*_cat_ min^−1^	*k*_cat_/*K*_m_ s^−1^mM^−1^
WT	137.6 ± 21.0	23.2 ± 0.7	2.8	217.0 ± 60.6	25.1 ± 2.4	1.9	174.3 ± 17.9	26.2 ± 0.6	2.5	349.8 ± 77.6	9.0 ± 0.3	0.4
XS2	387.9 ± 32.7	11.0 ± 0.4	0.47	490.8 ± 17.3	16.9 ± 2.6	0.6	404.6 ± 93.8	24.3 ± 2.4	1.0	1061.7 ± 21.1	6.5 ± 0.3	0.1
XS3	235.6 ± 40.2	29.8 ± 1.3	2.1	266.2 ± 41.1	14.8 ± 0.6	0.9	235.8 ± 52.8	33.9 ± 2.8	2.4	417.2 ± 10.2	9.0 ± 0.4	0.4
XS4	210.8 ± 85.3	30.7 ± 3.7	2.4	204.5 ± 16.3	22.3 ± 0.5	1.8	441.8 ± 22.1	50.8 ± 8.8	1.9	627.5 ± 75.0	6.1 ± 0.1	0.2
XS5	235.3 ± 52.5	22.5 ± 1.3	1.56	346.4 ± 11.2	13.0 ± 1.6	0.6	319.8 ± 9.9	20.0 ± 2.4	1.0	661.0 ± 93	7.8 ± 0.2	0.2
XS6	132.1 ± 29.1	21.9 ± 1.0	2.8	176.9 ± 35.9	20.9 ± 1.4	2.0	144.0 ± 22.9	25.0 ± 1.2	2.9	191.6 ± 33.6	9.0 ± 0.2	0.8

Data are presented as mean ± s.d. (n = 2).

To further understand the relationship between the structure of this loop and the enzyme catalytic properties, we designed and created mutant XS5 by changing the secondary structure of the loop to investigate its impact on the enzyme activity and substrate specificity. Considering EcHpaB completely lost its catalytic activity when the loop was simply replaced by a helix linker (data not show), we adopted a more flexible secondary loop structure to generate XS5 by using XS4 as the template (Table 1 and Fig. 5). Previous studies showed that the peptide chain tends to form random coil if it contains more Gly, Ser, Asn, Asp, Pro, His^29–31^. Therefore, the secondary structure of the β32-β33 loop was changed by replacing residues in section II of the loop (GlnValMet) with GlySerGly in order to generate a random coil structure. Swiss Model analysis showed that these changes could form a β-turn in the N-terminal region of section I of the loop followed by random coil structure in sections II and III (Fig. 5b). Compared with XS4, XS5 has smaller amino acid residues within section II, however, shows reduced catalytic activity towards *p*-coumaric acid, umbelliferone and resveratrol, with *k*_cat_ values reduced from 30.7 min^−1^, 22.3 min^−1^, 50.8 min^−1^ to 22.5 min^−1^, 13.0 min^−1^, 20.0 min^−1^, respectively. It should be noted that mutants XS4 and XS5 have identical amino acid sequences in sections I and III of the loop but completely different secondary structures of sections I and II, which led to the different observed catalytic capabilities. These results suggested that the flexibility of β32-β33 loop structure determined by the amino acid sequence may also have strong influence on the catalytic capability of EcHpaB by affecting substrate binding.

**Figure 7:**
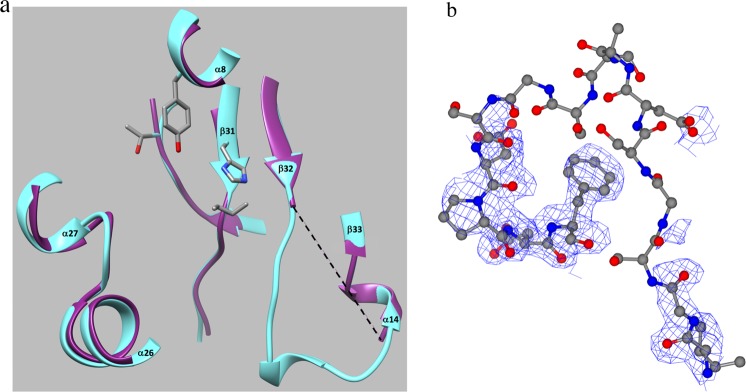
(**a**) A superposition (CHIMERA)^28^ of the EcHpaB and XS6 structures highlighting the active site (cyan: the active site of the EcHpaB and purple: active site of the XS6). The superposition gave an R.M.S.D of 0.323 Å for 509 Cα pairs. (**b**) A model of the XS6 β32-β33 loop structure (residues 206–220) showing the electron density observed in this region (CCP4MG)^47^. The model was generated by superposition^28^ of the native and XS6 EcHpaB structures with the coordinates for the EcHpaB loop residues 206–220 mutated (COOT)^43^ to correspond to residues in the XS6 mutant. The (2Fo-Fc) electron density map (contoured at 1 σ) for this region shows a lack of electron density for residues 208–216 of the β32-β33 loop. This missing density is consistent with a highly flexible loop which was the aim of the XS6 mutant design. (Color key: carbon-gray, nitrogen-blue, oxygen-red and electron density-blue).

In order to further test our hypothesis, mutant XS6 was designed and generated by employing a loop containing residues mainly Gly, Ser, and Asp, which are usually not involved in secondary structure formation. Swiss-model suggested that the β32-β33 loop of XS6 was a random coil (Fig. 5b). The results of *in vitro* enzyme assays revealed that XS6 had the almost same specificity constant values (*k*_cat_/*K*_m_) towards *p*-coumaric acid and umbelliferone as wild type EcHpaB and had even higher specificity constant values towards larger substrates such as resveratrol and naringenin compared with wild type EcHpaB. The *K*_m_ values towards *p*-coumaric acid, umbelliferone, resveratrol and naringenin decreased from 137.6 μM, 217.0 μM, 174.3 μM and 349.8 μM to 132.1 μM, 176.9 μM, 144.0 μM and 191.6 μM, respectively (Table 2), which indicated XS6 has improved affinity towards all the tested substrates, especially, the largest one, naringenin. In addition, we also conducted whole-cell bioconversion experiments to compare the *in vivo* catalytic performance of the mutants with wild type EcHpaB. The conversion rate of naringenin by XS6 was 2.95 mg/L/OD, representing a 56.1% increase compared with wild type HpaB, which was 1.89 mg/L/OD (Fig. S2). It is worthy to note that the β32-β33 loop of XS6 contains smaller amino acid residues and has random coli structure. The improved catalytic efficiency of XS6 towards large molecules further suggested that both smaller amino acid residues and the flexibility of this loop structure during substrate binding are beneficial for the enzyme to accept larger substrates. To further examine this, we determined the crystal structure of mutant XS6 (Table S2 and Fig. 7). Compared with wild type EcHpaB, the electron density of residues 208–216 in the loop connecting strands β32-β33 was missing in mutant XS6 (Fig. 7a, indicated by the dashed line), verifying that the loop in XS6 is highly flexible (Fig. 7b).

Overall, these results suggested that the specific secondary loop structure (random coli-β turn-random coli) covering the active site of EcHpaB is not the essential structural characteristic to its broad substrate specificity. Instead, a flexible loop that enables the entrance and stable binding of substrates into the active site is the key factor to the enzyme catalytic versatility. Such flexibility is determined by the amino acid composition in the loop.

## Discussion

In this study, we determined the crystal structure of EcHpaB apoenzyme by X-ray crystallography. It allowed us to understand its structure-activity relationship. Compared with the homologous hydroxylase from *T. thermophilus* HB8*, EcHpaB exhibits higher activity especially towards large substrates. Notable differences observed in the 11-residue loops connecting strands β32 and β33 between the *E. coli* and *T. thermophilus* enzymes prompted us to investigate the impacts of changing the amino acid composition and secondary structure of the loop on the catalytic properties of the enzyme.

In order to better understand the roles of this loop in catalytic activity and substrate specificity, seven mutants with different amino acid sequences and hypothesized secondary structures for the β32-β33 loop were constructed. Their enzymatic activities towards four substrates with different molecular shapes and sizes were measured by *in vitro* enzyme assays. XS6, a mutant with smaller amino acids residues and random coil structures in the loop showed higher activity towards larger substrates such as resveratrol and naringenin compared with wild type EcHpaB. Notably, the catalytic activity of XS6 towards naringenin increased by 56.1%. We further determined the crystal structure of XS6. The sequence and structural analyses indicated the increased activities of XS6 towards larger substrates are most likely caused by increased loop flexibility that is beneficial to substrate entrance and binding.

In general, the catalytic pockets of enzymes are usually quite rigid^32^. Only few amino acid residues in the catalytic pockets can be altered to achieve improved or new functions^32,33^. However, extensive modifications of the amino acid residues involved in substrate binding in the catalytic pockets tend to completely inactive the enzymes^34,35^. Interestingly, in our study, the entire β32-β33 loop of EcHpaB that is responsible for substrate specificity demonstrates great plasticity and exhibits strong tolerance towards extensive mutagenesis without causing substantial activity loss. This unique feature offers a great opportunity to reprogram the loop sequence and structure for generation of HpaB mutants with improved performance on more non-natural substrates. Overall, the crystal structure of EcHpaB determined in this study provides a solid foundation for further protein engineering studies of this enzyme.

## Materials and Methods

### Strains, plasmids and media

*E. coli* XL-1Blue was used to construct and propagate plasmids in this research. *E. coli* BW25113 (F’) was recruited as the host to overexpress proteins for enzyme assays and to do the whole-cell conversion. Luria Bertani (LB) medium was employed to support *E. coli* cells growth for plasmid construction, protein overexpression and inoculum preparation. M9 medium (M9Y) was used to do whole-cell biocatalysis. M9Y is composed of 20 g/L glucose, 5 g/L yeast extract, 1 g/L NH_4_Cl, 6 g/L Na_2_HPO_4_, 3 g/L KH_2_PO_4_, 0.5 g/L NaCl, 246.5 mg/L MgSO_4_·7H_2_O and 14.7 mg/L CaCl_2_·2H_2_O. Plasmid pZE12-luc was used for expression of wild type EcHpaB as well as the mutated EcHpaBs. Plasmid pCS27 was adopted to express HpaC. A final concentration of 0.5 mM IPTG was introduced into the media after the cell mass optical density (OD_600_) reached 0.4–0.6 during protein overexpression and whole-cell biocatalysis. The strains and plasmids used in this research are listed in Table S1.

### Crystallization and X-ray data collection

Structure determination of the EcHpaB apo enzyme. Crystals of EcHpaB were grown by the microbatch-under-oil method^36^ at 291 K using 2 μL drops containing equal volumes of the protein and a precipitant cocktail containing 0.08 M Sodium Cacodylate pH = 6.5, 0.16 M Magnesium Acetate tetrahydrate, 16% Polyethylene Glycol 8000, and 20% v/v Glycerol. Crystals appeared within 5 days and grew to usable size in 9–10 days.

For data collection, a crystal measuring 80 × 50 × 10 microns was harvested^37^ from the well and briefly (5–10 sec) immersed in a drop of cryoprotectant solution containing the above precipitant cocktail with 30% (v/v) glycerol^38^. The cryoprotected crystal was then flash cooled in liquid nitrogen and stored at cryogenic temperatures for data collection.

A data set to 2.37 Å resolution was collected at 100 K on beamline 22ID, SER-CAT, Advanced Photon Source, Argonne National Laboratory (www.ser-cat.org) using a 50-micron beam, a MAR MX300 CCD detector and 0.979 Å X-rays. A total of 200 one-degree images were recorded using a crystal-to-detector distance of 330 mm and an exposure time of 1 second. The data were indexed, integrated and scaled using HKL-2000^39^.

The structure was solved using the automated CCP4 BALBES^40^ web service. BABLES identified ten possible search models based on sequence homology to EcHpaB. An ensemble search model was then generated based residues 2–477 of chain A of the oxygenase component (HpaB) of 4-hydroxyphenylacetate 3-monooxygenase (PDB entry 2YYG)^16^ from *Thermus thermophiles* HB8*, the closest PDB sequence homologue (30% identity). BALBES gave single molecular replacement solution containing one EcHpaB tetramer in the asymmetric unit. Subsequently, 10 cycles of automated model building using ARP/wARP^41^ verified that MR solution was correct building 2026 residues out of 2112 residues (96% complete) in the asymmetric unit and gave R and R_free_ values of 16.4% and 22.4%, respectively. The model was further improved using iterative rounds of validation (MolProbity)^42^ model building (COOT)^43^ and refinement using PHENIX^44^ employing torsion angle NCS restraints and TLS. As outlined in Table S2 the refinement converged to give R and R_free_ values of 0.1583 and 0.1932, respectively and had good stereochemistry, with RMSDs from ideality of 0.008 Å for bond lengths and 1.04° for bond angles. The final refined model contains four peptide chains, 2072 residues (98% complete), 850 solvent molecules modeled as water and 3 acetate ions. The coordinates and structure factors have been deposited in the Protein Data Bank as entry 6EB0^45^.

### Structure determination of the EcHpaB XS6 mutant

Crystals of the EcHpaB XS6 mutant were grown by the vapor diffusion hanging drop method at 291 K using 1:1 mixture of protein and a precipitant cocktail containing 1.0 M Succinic acid pH 7.0, 0.1 M HEPES, pH 7.0, and 1% w/v Polyethylene glycol monomethyl ether 2,000. Crystals appeared within 3–4 days and grew to usable size in 5–6 days.

For data collection, a crystal of the mutant enzyme measuring 80 × 50 × 15 microns was harvested and cryoprotected with 4 M Trimethylamine N-oxide dihydrate. A data set to 1.94 Å resolution was collected at 100 K on beamline 22ID using a Rayonix MX300HS CCD detector and 1.0 Å X-rays. A total of 720 (0.25° oscillation) images were collected using a 200 mm crystal-to-detector distance and an exposure time of 0.25 seconds. The data were indexed, integrated and scaled using HKL2000.

The structure of apo EcHpaB XS6 mutant was solved by molecular replacement using PHASER (CCP4) with the refined apo EcHpaB structure (PDB entry 6EB0) used as the search model. PHASER gave single MR solution containing two polypeptide chains in the asymmetric unit. The model was further improved using iterative rounds of validation (MolProbity)^42^ model building (COOT)^43^ and refinement using PHENIX^44^ employing torsion angle NCS restraints and TLS. The final refinement converged to give R and R free values of 0.1516 and 0.1661, respectively (Table S2) and had good stereochemistry, with RMSDs are 0.007 Å for bond lengths and 0.939° for bond angles. The final refined model contains two peptide chains residues 2–206, 218–519 and 960 solvent molecules modeled as water. In addition, the crystal structure revealed 8 Trimethylamine N-oxide (TMO) molecules from cryoprotectant. In both molecules, residue 1, the β32-β33 loop residues 208–216 and residue 520 appear to be disordered since they show no electron density. Since XS6 was designed to give the β32-β33 loop increased flexibility, the absence of electron density for these residues, a hallmark of structural flexibility, is not surprising. The coordinates and structure factors for the EcHpaB XS6 mutant have been deposited in the Protein Data Bank as entry 6B1B^45^.

### Modelling of the EcHpaB active site and substrate docking

Sequence comparisons using ENDscript 2.0^46^ showed that key active site residues were conserved between the EcHpaB and TtHpaB enzymes (Fig. 3a). This suggested that we could use the superposition of the Apo EcHpaB (PDB entry 6EB0) and substrate and cofactor bound TtHpaB (PDB entry 2YYJ) structures to make a crude model of the EcHpaB active site. To do this the 6EB0 and 2YYJ models were superimposed using the CHIMERA *MatchMaker* utility^28^. The superposition gave an RMSD of 1.3 Å for 403 Cα pairs. The aligned 2YYJ model was then saved in the 6EB0 reference frame. Next, the 6EB0 PDB entry was edited and residues 206–226 were replaced with residues 193–213 from the aligned 2YYJ model. The residue range chosen ensured maximum overlap of the 2YYJ coordinates with the 6EB0 structure at the N and C terminal junctures. The 4HP substrate and FAD cofactor from the aligned 2YYJ model were then appended to the 6EB0 coordinate set. COOT^43^ was then used to renumber the inserted residues and to mutate their sequences to correspond to the correct EcHpaB sequence. The resulting model although crude showed that the conserved active site residues were in position to bind substrate and FAD with small main chain/side chain movements and that the grafted β32-β33 loop placed the conserved Ser 210 in close proximity to the carboxyl tail of the 4HP substrate.

### Enzyme assays

The procedure of HpaB enzyme assays was created by Louie and modified in this study^24^. Briefly, the kinetic assay reaction mixture contains 20 mM KPi buffer (pH = 7.0), 10 μM FAD, 1 mM NADH, 500 nM purified wild type or mutated HpaB protein, 500 nM purified HpaC protein, the concentration of substrates varied from 0 to 1000 μM. The mixture was incubated at 30 °C in shaking bath for 15 min to 30 min (for different mutations). 25 μL HCl (40%) was used to stop the reaction. The product generation and substrate consumption rates were measured by HPLC. Apparent kinetic parameters were determined by non-linear regression of the Michaelis–Menten equation by OriginPro8.5^TM^.

## Supplementary information

Supporting information
