# Supplementary material for: Structural Insights into Catalytic Versatility of the Flavin-dependent Hydroxylase (HpaB) from Escherichia coli
Source: Sci Rep. 2019 May 8;9:7087. doi: 10.1038/s41598-019-43577-w (PMC6506529; doi:10.1038/s41598-019-43577-w)
Supplement: Supplementary file 1 — Supporting information [file 41598_2019_43577_MOESM1_ESM.pdf]

Supporting Information

Structural Insights into Catalytic Versatility of the Flavin-dependent Hydroxylase (HpaB) from *Escherichia coli*

Xiaolin Shen[1,2,#], Dayong Zhou[3,#], Yuheng Lin[4,#],

Jia Wang[1,2], Shuaihua Gao[1,2], Palani Kandavelu[3], Hua Zhang[3], Ruihua Zhang[4],

Bi-Cheng Wang[3], John Rose[3,*], Qipeng Yuan[1,2,*], Yajun Yan[4,*]

[1] State Key Laboratory of Chemical Resource Engineering, Beijing University of Chemical Technology, Beijing 100029, China

[2] Beijing Advanced Innovation Center for Soft Matter Science and Engineering, Beijing University of Chemical Technology, Beijing 100029, China

[3] Department of Biochemistry & Molecular Biology, The University of Georgia, Athens, Georgia 30602, USA

[4] College of Engineering, The University of Georgia, Athens, GA 30602, USA

[*]Corresponding Authors: John Rose, Qipeng Yuan and Yajun Yan

John Rose

B204B Life Sciences, The University of Georgia, Athens, GA 30602, USA

Email: jprose@uga.edu

Qipeng Yuan

15# Beisanhuan East Road, Chaoyang District, Beijing 100029, China

Email: yuanqp@mail.buct.edu.cn

Yajun Yan
146 Riverbend Research Lab South, The University of Georgia, Athens, GA 30602, USA
Email: yajunyan@uga.edu

[#]Xiaolin Shen, Dayong Zhou and Yuheng Lin contributed equally to this work.

Supporting Materials and methods

Expression and Purification of apo and mutant EcHpaB

Expression and purification of the EcHpaB apo enzyme. The expression plasmid pXS8 was constructed by inserting *E. coli hpaB* into the BamHI/NdeI restriction sites of the pETDuet-1 vector. *E. coli* strain BL21 Star (DE3) was then transformed with plasmid pXS8 containing an N-terminal 6×His-tag to aid in purification. A fresh colony was inoculated into 50 mL LB medium containing 100 μg/mL ampicillin and grown aerobically at 37 °C overnight. The whole overnight culture was then used to inoculate 1 L of LB medium supplemented with 100μg/ml ampicillin and grown at 37 ºC with shaking (250 rpm). When OD_{600} reached 0.6, the culture was induced with 0.5 mM IPTG and cultivated at 30°C for an additional 3 hours. Cells were harvested by centrifugation at 6000× *g* for 15 min at 4 º C and resuspended in 30 ml lysis buffer (20 mM phosphate buffer, pH 7.4, 500 mM NaCl, 20 mM imidazole, 10 μg/mL phenylmethylsulfonyl fuoride). The cells were lysed by sonication on ice and cleared by centrifugation at 25,000× *g* for 30 minutes. The supernatant was loaded onto a HisTrap HP column (5 ml, GE Healthcare) pre-equilibrated with binding buffer (20 mM phosphate buffer, pH 7.4, 500 mM NaCl, 20 mM imidazole) and connected to AKTAprime plus (GE Healthcare). The column was washed with 50 ml of binding buffer and EcHpaB proteins were eluted with a linear imidazole concentration gradient (20 to 500 mM). The resulting EcHpaB eluents were further purified on a preparative gel filtration Superdex 200 10/60 column (GE Healthcare) pre-equilibrated with 20 mM Tris-HCl, pH 7.4, 50 mM NaCl, 1 mM DTT, and the fractions containing purified EcHpaB were pooled and concentrated to approximately 14 mg/ml for crystallization.

Expression and purification of the EcHpaB XS6 mutant enzyme. The expression and purification of EcHpaB XS6 mutant employed the same procedures as used for EcHpaB apo enzyme with the exception that the purified mutant protein was concentrated to approximately 10 mg/mL for crystallization.

HPLC analysis

The chemicals *p*-coumaric acid, umbelliferone, resveratrol, naringenin, caffeic acid, esculetin, piceatannol and eriodictyol in the processes of whole-cell catalysis and enzyme assays were quantitatively analyzed by Dionex Ultimate 3000 (Ultimate 3000 Photodiode Array Detector) with a reverse-phase ZORBAX SB-C18 column. A Methanol-water (containing 0.2 % trifluoroacetic acid) gradient system at a flow rate of 1 mL/min was used to separate the compounds. The HPLC program was as follows: 10 to 70 % methanol for 15min, 70 to 10 % methanol for 1 min, and 10 % methanol for 4 min. UV absorbance at 280, 274, 308 and 323 nm were quantified for the eight compounds mentioned above, respectively. The enzyme assays data and whole-cell catalysis data shown in this study were collected from duplicate or triplicate experiments.

Table S1. Strains and plasmids used in this study.

Strain	Genotype	Reference
E. coli BW25113/F'	*F+, Δ(araD-araB)567, ΔlacZ4787(::rrnB-3),* λ^-*, rph-1, Δ(rhaD-rhaB)568, hsdR514 [F' proAB lacIqZDM15Tn10 (TetR)]*	Yale CGSC
E. coli XL-1 Blue	*recA1 endA1gyrA96thi-1hsdR17supE44relA1lac [F' proAB lacIqZDM15Tn10 (TetR)]*	Stratagene
E. coli BL21 Star (DE3)	F^-*ompT hsdS*$_B$ *(*$r_B^- m_B^-$*) gal dcm (DE3)*	Invitrogen

Plasmid	Description	Reference
pZE12-luc	pLlacO1; luc; *ColE1 ori*; *Amp*^{R}	[43]
pCS27	pLlacO1; *p15A ori; Kan*^{R}	[44]
pETDuet-1	two T7 promoters; two MCS; *pBR322 ori*; *Amp*^{R}	Novagen
pXS2	Variant *xs2* cloned into pZE12-luc	This study
pXS3	Variant *xs3* cloned into pZE12-luc	This study
pXS4	Variant *xs4* cloned into pZE12-luc	This study
pXS5	Variant *xs5* cloned into pZE12-luc	This study
pXS6	Variant *xs6* cloned into pZE12-luc	This study
pXS7	Wildtype *hpaB* from *E. coli* BL21* (DE3) cloned into pZE12-luc	This study
pXS8	Wildtype *hpaC* from *E. coli* BL21* (DE3) cloned into pCS27	This study
pXS9	Wildtype *hpaB* from *E. coli* BL21* (DE3) cloned into pETDuet-1	This study

Table S2. Data collection and refinement statistics.

	Apo EcHpaB	Apo EcHpaB XS6 Mutant
**Wavelength (Å)**	0.979	1.0
**Resolution range (Å)**	46.36 - 2.37 (2.455 - 2.37)	44.53-1.94 (2.01-1.94)
**Space group**	C 1 2 1	$P4_322$
**Unit cell (Å)**	179.41 93.73 142.34 β=108.23	100.45 100.45 336.45
**Total reflections**	387609	2194280
**Unique reflections**	90432 (8497)	127481 (12468)
**Multiplicity**	4.3 (4.2)	17.2 (16.8)
**Completeness (%)**	99.28 (93.48)	99.89 (99.48)
**Mean I/sigma(I)**	15.81 (3.27)	29.736 (8.45)
**Wilson B-factor**	35.30	18.77
**R-merge**	0.113 (0.475)	0.120 (0.698)
**R-meas**	0.151 (0.556)	0.113 (1.523)
**R-pim**<br>**CC1/2**	0.030 (0.072)<br>0.839	0.030 (0.178)<br>0.960
**CC***	0.955	0.990
**Reflections used for R-free**	4400	1999
**R-work**	0.1583 (0.1979)	0.1516 (0.1748)
**R-free**	0.1932 (0.2704)	0.1661 (0.2058)
**Non-hydrogen atoms**	17132	9230
**macromolecules**	16370	8129
**ligands**	14	40
**water**	850	1061
**Protein residues**	2072	1019
**RMS bond distances (Å)**	0.008	0.007
**RMS bond angles (Å)**	1.04	0.939
**Ramachandran favored (%)**	97	97.33
**Ramachandran allowed (%)**	2.81	2.27
**Ramachandran outliers (%)**	0.19	0.40
**Clash score**	9.02	4.9
**Average B-factor**	35.80	22.94
**macromolecules**	35.70	21.09
**ligands**	49.10	38.86
**solvent**	38.40	36.52

Statistics for the highest-resolution shell are shown in parentheses.

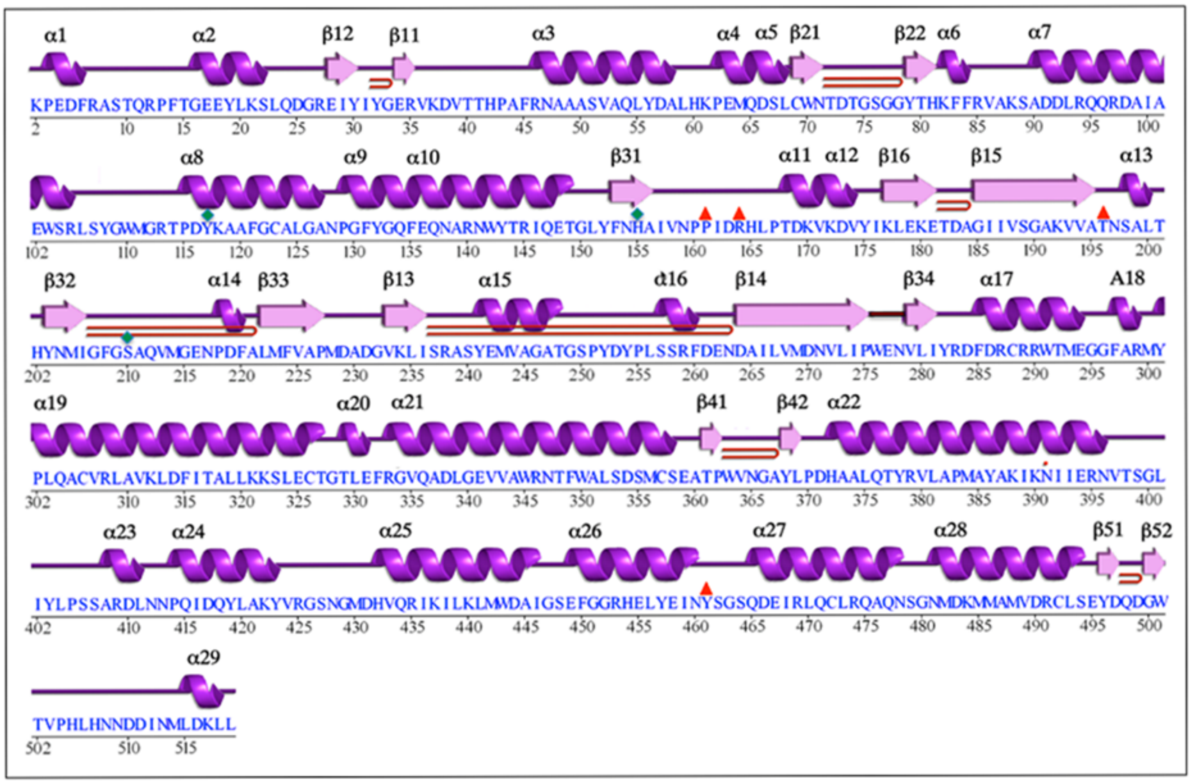

Figure S1. A wiring diagram showing the secondary structural elements (helices, strands and turns) found in the EcHpaB PDB entry 5UM5 mapped onto the EcHpaB amino acid sequence. Helices, strands and turns are denoted by purple coils, pink arrows and red hairpins, respectively. Residues predicted (based on modeling) to interact (hydrogen bonding) with FAD are denoted by red triangles. Residues predicted to interact with substrate are denoted by green diamonds. Secondary structural features are named using α to denote a helix and β to denote a strand. Helices are numbered sequentially from the N- to C-terminus. Strands are numbered by the β ladder found in the PDB entry with the first integer representing the sheet identifier and the second integer representing the strand in the ladder. For example, β12 corresponds to β sheet 1 strand 2.

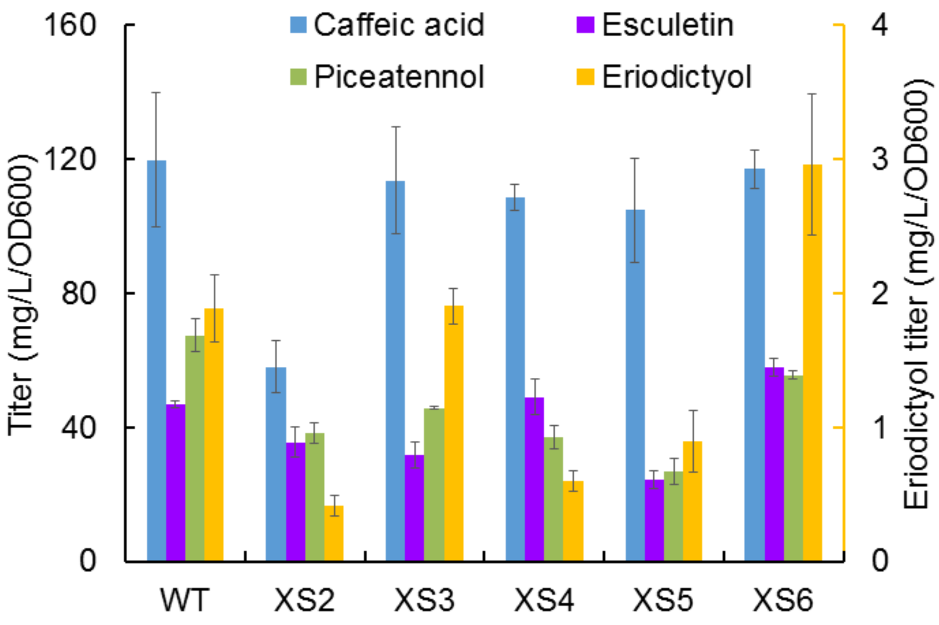

Figure S2. Results of whole cell biocatalysis experiments toward *p*-coumaric acid, umbelliferone, resveratrol and naringenin using wildtype HpaB and its variants. All data points are reported as mean ± s.d. from three independent experiments.
